# *Klf4*,* Klf2*, and *Zfp148* activate autophagy‐related genes in smooth muscle cells during aortic aneurysm formation

**DOI:** 10.14814/phy2.14058

**Published:** 2019-04-25

**Authors:** Morgan Salmon, Michael Spinosa, Zendra E. Zehner, Gilbert R. Upchurch, Gorav Ailawadi

**Affiliations:** ^1^ Department of Surgery University of Virginia School of Medicine Charlottesville Virginia USA; ^2^ Department of Biochemistry Virginia Commonwealth University Medical Center Richmond Virginia USA; ^3^ Department of Surgery University of Florida Gainesville Florida USA; ^4^ The Robert M. Berne Cardiovascular Research Center University of Virginia School of Medicine Charlottesville Virginia USA

**Keywords:** Aortic Aneurysm, autophagy, smooth muscle cells, *Zfp148*, *Klf2*, *Klf4*

## Abstract

Abdominal aortic aneurysms (AAAs) are a progressive dilation of the aorta that is characterized by an initial influx of inflammatory cells followed by a pro‐inflammatory, migratory, proliferative, and eventually apoptotic smooth muscle cell phenotype. In recent years, the mechanisms related to the initial influx of inflammatory cells have become well‐studied; the mechanisms related to chronic aneurysm formation, smooth muscle cell apoptosis and death are less well‐characterized. Autophagy is a generally believed to be a protective cellular mechanism that functions to recycle defective proteins and cellular organelles to maintain cellular homeostasis. Our goal with the present study was to investigate the role of autophagy in smooth muscle cells during AAA formation. Levels of the autophagy factors, Beclin, and LC3 were elevated in human and mouse AAA tissue via both qPCR and immunohistochemical analysis. Confocal staining in human and mouse AAA tissue demonstrated Beclin and LC3 were present in smooth muscle cells during AAA formation. Treatment of smooth muscle cells with porcine pancreatic elastase or interleukin (IL)‐1*β* activated autophagy‐related genes in vitro while treatment with a siRNA to *Kruppel‐like transcription factor 4* (*Klf4*), *Kruppel‐like transcription factor 2* (*Klf2*) or *Zinc‐finger protein 148* (*Zfp148*) separately inhibited activation of autophagy genes. Chromatin immunoprecipitation assays demonstrated that *Klf4*,* Klf2*, and *Zfp148* separately bind autophagy genes in smooth muscle cells following elastase treatment. These results demonstrate that autophagy is an important mechanism related to Klfs in smooth muscle cells during AAA formation.

## Introduction

Abdominal aortic aneurysms (AAA) remain a major healthcare concern in the United States because they are a gender‐specific disease that targets men 4:1 over women (United States Centers for Disease Control and Prevention, O. o. S. a. P., and the National Center for Injury Prevention and Control, WISQARS 2007; Kent et al. 2010; Hoyert and Xu 2012; Bhak et al. 2014). AAAs are especially concerning as they are asymptomatic, difficult to diagnose, and rupture results in a high‐mortality rate, resulting in AAAs being the 15th leading cause of death in the aging population. Therefore, as Western populations continue to age, these numbers will continue to increase until better methods to diagnose and treat aneurysms are identified (United States Centers for Disease Control and Prevention, O. o. S. a. P., and the National Center for Injury Prevention and Control, WISQARS 2007; Kent et al. 2010; Hoyert and Xu 2012; Bhak et al. 2014). Even more concerning, AAAs results in more than 15,000 surgical procedures annually as there is currently no medical treatment strategy for AAA growth rates (United States Centers for Disease Control and Prevention, O. o. S. a. P., and the National Center for Injury Prevention and Control, WISQARS 2007; Kent et al. 2010; Hoyert and Xu 2012; Bhak et al. 2014). Aortic aneurysms have historically been characterized by an early elevation of pro‐inflammatory cytokines such as IL‐1*β*, monocyte chemotractant protein 1 (MCP‐1), IL‐6, tumor necrosis factor alpha (TNF*α*), interferon gamma (IFN*γ*), IL‐23, IL‐17 (Moehle et al. 2011; Sharma et al. 2012; Johnston et al. 2013). As a result, the pathways and mechanisms of action of many of these cytokines on AAA formation have been at least initially examined. In many cases, it is believed that these processes are associated with encouraging elastin and collagen degradation. Later, smooth muscle cell apoptosis, necrosis, and autophagy are coupled with further collagen and elastin degradation and lead to the chronic, thrombotic process seen in most human AAAs (Holmes et al. 1996; Thompson et al. 1997). The effects of smooth muscle cell apoptosis and necrosis accompanied by extracellular matrix degradation is believed to be the major processes leading to the weakening of the aortic wall and could be one of the major causes of AAA rupture; however, the mechanisms that regulate AAA rupture remain unknown (Henderson et al. 1999; Carmo et al. 2002; Middleton et al. 2007). Our laboratory was the first to demonstrate that Smooth muscle cell (SMC) phenotypic switching was an early event in aortic aneurysm formation and that the Kruppel‐like transcription factors (*Klf4 [Kruppel‐like transcription factor 4]*,* Klf2 [Kruppel‐like transcription factor 2]* and *Zfp148 [Zinc‐finger protein 148]*) plays a role in SMC phenotypic modulation (Klf2; M. Salmon unpubl. obs.) (Ailawadi et al. 2009; Salmon et al. 2013, 2019). The Kruppel‐like transcription and zinc‐finger transcription factor families are transcriptional activators and/or repressor that regulate multiple disease processes such as atherosclerosis and AAA formation via modulation of key down‐stream inflammatory processes. Currently there is currently little information, besides our own studies, to describe the mechanism of smooth muscle cell phenotypic switching in the context of AAA formation (Ailawadi et al. 2009).

Macroautophagy (or autophagy) is the major intracellular recycle system and processes defective organelles and proteins for recycling to help maintain cellular homeostasis. Studies have shown that during stress, autophagy functions to maintain the integrity of the cell and to encourage the return to homeostasis (Nussenzweig et al. 2015; Ramadan et al. 2017a). Therefore, it is commonly believed that autophagy mainly functions to maintain cell function and to prevent cell death and senescence, which are increased during such process as atherosclerosis or AAA formation (Nussenzweig et al. 2015; Ramadan et al. 2017a). To that end, recent studies investigated the role of endothelial‐specific ATG7 deficiency in mice and demonstrated an increase in atherosclerotic plaque development (Yau et al. 2017). However, few studies have investigated the role of autophagy in AAA formation and in smooth muscle cells during the formation of the disease (Ramadan et al. 2015, 2017a,b). The few studies have examined the role of inhibition of autophagy using such treatment therapies as chloroquine but with limited success (Ramadan et al. 2015). Therefore, the goal of this study was determine the role of autophagy in smooth muscle cells and whether the Klf or Sp transcription factor families could be responsible for activation of autophagy‐related genes during AAA formation (Salmon et al. 2013).

## Materials and Methods

### Murine elastase model

Eight to 12‐week male mice (*n* = 12/group) were injected with intraperitoneal ketamine solution, elastase perfused and harvest as previously described by Johnston et al. (Salmon et al. 2013; Johnston et al. 2014a,b). The aortas (or aneurysms, when present) were harvested, and either: (1) snap frozen in liquid nitrogen for analyses by real‐time polymerase chain reaction (qPCR) or protein extraction, or (2) incubated overnight for histology or immunohistochemistry. Animal care and use were in accordance with the *Guide for the Care and Use of Laboratory Animals*. The animal protocol was approved by the University of Virginia Institutional Animal Care and Use Committee (#3634) in compliance with the Office of Laboratory Animal Welfare.

### Human tissue harvest

Normal abdominal aortas were obtained from transplant donors and aneurysmal aortas were taken from patients undergoing elective open AAA repair. Aortas from patients with known collagen vascular disease were excluded. Aortic tissue specimens were explanted, placed on ice, and then immediately snap‐frozen in liquid nitrogen or placed in 10% formalin for histological processing (Salmon et al. 2013; Johnston et al. 2014a,b). Collection of human tissue was approved by the patient's written consent in compliance with the University of Virginia's Human Subjects Review Committee (HSR #13178).

### mRNA isolation and qPCR analysis

mRNA was extracted from frozen aortic tissue samples with TRIzol reagent (Invitrogen, Life Technologies, Grand Island, NY) (Salmon et al. 2009, 2012). Using isolated mRNA from the mouse aortas and aortic smooth muscle cells, cDNA was synthesized using iScript cDNA synthesis kit (Bio‐Rad, Hercules, CA). Real‐time reverse transcription PCR (RT‐PCR) was performed using Sensifast SYBR Supermix (Bioline, Taunton, MA) with human primers as described in Table 1 and mouse primers as described in Tables 2 and 3. Target DNA was analyzed with Bio‐Rad CFX Manager software (Bio‐Rad, Hercules, CA) to obtain melt curves and takeoff values. Levels of mRNA were standardized either with U6 or with 18s mRNA, which served as a housekeeping genes for comparison. All experiments were run three times in triplicate unless otherwise mentioned.

**Table 1:** Human primers for qPCR

*beclin‐1*‐forward	5′‐ACCGTGTCACCATCCAGGAA‐3′
*beclin‐1*‐reverse	5′‐GAAGCTGTTGGCACTTTCTGT‐3′ (Morikawa et al. 2015)
*LC3‐II* For	5′‐GAGCAGCATCCAACCAAA‐3′
*LC3‐II* Rev	5′‐CGTCTCCTGGAGGCATA‐3′
Human 18S For	5′‐GGCCCTGTAATTGGAATGAGTC‐3′
Human 18s Rev	5′‐CCAAGATCCAACTACGAGCTT‐3′

Autophagy‐related gene primers for qPCR.

**Table 2:** Zinc finger transcription factor qPCR

*Klf1* For	5′‐ATGAGGCAGAAGAGAGAGAGGA‐3′
*Klf1* Rev	5′‐AAATCCTGCGTCTCCTCAGA‐3′ (Sorolla et al. 2015)
*Klf2 For*	5′‐CGCCTCGGGTTCATTTC‐3′
*Klf2 Rev*	5′‐AGCCTATCTTGCCGTCCTTT‐3′ (Takada et al. 2011)
*Klf3* For	5′‐TGCAAGAGAACCATCCTTCC‐3′
*Klf3* Rev	5′‐GGTGCATTTGTACGGCTTTT‐3′ (Himeda et al. 2010)
*Klf4* For	5′‐CTTTCCTGCCAGACCAGATG‐3′ (Liu et al. 2005)
*Klf4* Rev	5′‐GGTTTCTCGCCTGTGTGAGT‐3′ (Liu et al. 2005)
*Klf5* For	5′‐ACCAGACGGCAGTAATGGACAC‐3′
*Klf5* Rev	5′‐ATTGTAGCGGCATAGGACGGAG‐3′ (Lin et al. 2010)
*Klf6* For	5′‐GGACCAAATTCATTCTAGCTCGGG‐3′
*Klf6* Rev	5′‐AGGCGTCGCCATTACCCTTG‐3′ (Nakamura et al. 2004)
*Klf7* For	5′‐CCTGGCAGCAGACATGCCTTGA‐3′
*Klf7* Rev	5′‐AGGCGCCGGAAGCTCTCCTC‐3′
*Klf8* For	5′‐TGGATGTCCGAATTAAATCAGAAA‐3′
*Klf8* Rev	5′‐GAAGGATCTCTGGTCGGAACAG‐3′ (Funnell et al. 2013)
*Klf9* For	5′‐GCCGCCTACATGGACTTCG‐3′
*Klf9* Rev	5′‐GGTCACCGTGTTCCTTGGT‐3′
*Klf10* For	5′‐AGCTGCGACTGGAAGTCTCA‐3′
*Klf10* Rev	5′‐CCTCGGAGGTATCAGACACTG‐3′
*Klf11* For	5′‐CATGGACATTTGTGAGTCGATCC‐3′
*Klf11* Rev	5′‐CCTTTGGTAGATCAGGTGCAG‐3′
*Klf12* For	5′‐GTCAAAACCGAGCTTGTGGAA‐3′
*Klf12* Rev	5′‐GGGCTCCCCTTTCACATTATTT‐3′
*Klf13* For	5′‐CCTCAGACAAAGGGGTCGG‐3′
*Klf13* Rev	5′‐GTAGTGGCACTTGTGCTTCC‐3′
*Klf14* For	5′‐CTCCGTGTGCCTCAACTAGC‐3′
*Klf14* Rev	5′‐CAGGCGCATCCAGGATAGC‐3′
*Klf15* For	5′‐GGCAGTGGAGGTATTGGAGAT‐3′
*Klf15* Rev	5′‐GGTCCCTGCTACCGTTCTCT‐3′
*Klf16* For	5′‐AGCATCCTGGCCGATCTGA‐3′
*Klf16* Rev	5′‐GTGCGAAGACTTGTAATAGGCT‐3′
*Klf17* For	5′‐AATAAGGAACAGGCTATGCACC‐3′
*Klf17* Rev	5′‐GTGGCTGATGAAATCCGCTG‐3′
Sp1 For	5′‐TGAGGCATTAATGTGCTTGG‐3′
Sp1 Rev	5′‐AAATGCTGATCAAAGGGTGG‐3′ (Salmon and Zehner 2009)
*Sp2* For	5′‐CCAGCCTACCCCAAGGAAAC‐3′
*Sp2* Rev	5′‐GGGAGCCCTGAATCTGAAGTAT‐3′ (Kim et al. 2010)
*Sp3* For	5′‐TGCCAACATCCTCTTCATCA‐3′
*Sp3* Rev	5′‐CAATTTGGGCTTGACTGGTT‐3′ (Salmon and Zehner 2009)
*Sp4* For	5′‐TTGCAGCAAGGCCAGCAGACC‐3′
*Sp4* Rev	5′‐GCTTCTTCTTTCCTGGTTCACTGCT‐3′ (Nair et al. 2016)
U6 For	5′‐CTCGCTTCGGCAGCACA‐3′ (Salmon and Zehner 2009)
U6 Rev	5′‐AACGCTTCACGAATTTGCGT‐3′ (Salmon and Zehner 2009)
*Zfp148* For	5′‐TCCAAACCACTGATTCTTCTCTT‐3′ (Salmon et al. 2009)
*Zfp148* Rev	5′‐AGTTCTCTCCCCTCCCCCT‐3′ (Salmon et al. 2009)
*Zfp281* For	5′‐GCACCACCGCGATGTATTACT‐3′
*Zfp281* Rev	5′‐CCTTTTTGACGTTAGCGTCCTG‐3′ (Huang et al. 2017)

Mouse primers for zinc finger transcription factor qPCR experiments. Primer sequences for mouse Klf9‐17 were obtained from https://pga.mgh.harvard.edu/cgi-bin/primerbank.

**Table 3:** Autophagy factor qPCR primers

*SM α‐actin*‐For	5′‐AGTCGCCATCAGGAACCTCGAG‐3′
*SM α‐actin*‐Rev	5′‐ATCTTTTCCATGTCGTCCCAGTTG‐3′
*SMMHC*‐For	5′‐CAGTTGGACACTATGTCAGGGAAA‐3′
*SMMHC*‐Rev	5′‐ATGGAGACAAATGCTAATCAGCC‐3′ (Liu et al. 2003)
*Epg‐2* For	5′‐TCGTCAGCAGAGGATCAAGA‐3′
*Epg‐2* Rev	5′‐GCCAGCATTTTGTCCAAGTT‐3′
*Atg‐2* For	5′‐CTCAACCACATGGTGTCGTC‐3′
*Atg‐2* Rev	5′‐CATCGGTATGGAAAGTAACACCA‐3′
*Epg‐4* For	5′‐TTCAGGAGCCTGTATGAGAGC‐3′
*Epg‐4* Rev	5′‐AGCGCAGAAATGAGAGTTCC‐3′
*Unc‐51* For	5′‐AAAAGGGCATCGTACATCGT‐3′
*Unc‐51* Rev	5′‐ATTTTGGGTGCGGGAGTT‐3′
*Vps34* For	5′‐AACCCTGTCAGAAGGTTGAATC‐3′
*Vsp34* Rev	5′‐TGACGAGCAAGTTGAGAGGA‐3′
*Atg16.1* For	5′‐CAGAAGTTGCTTTAGAAGAAAAACG‐3′
*Atg16.1* Rev	5′‐TTTTGTGTCCTTGTCGGTGA‐3′
*Epg‐1/atg‐13* For	5′‐ATCCAGCAAATGGAACCAAG‐3′
*Epg‐1/atg‐13* Rev	5′‐TGGAGTTGATTTTGGAGAATTG‐3′
*Pha‐4* For	5′‐CTACACCAACGGAGTATACCAGAA‐3′
*Pha‐4* Rev	5′‐GGAGAACGTGTGAATTGGAGA‐3′
*Atg‐7* For	5′‐TCTTGCTATTTCTGCAGTGATGT‐3′
*Atg‐7* Rev	5′‐GTTCCTGGTCGTGCAACAG‐3′
*Atg‐9* For	5′‐GGTCTTCACGATGAGAGTATTATCC‐3′
*Atg‐9* Rev	5′‐TGCATGTTGAAGCTTGACG‐3′
*Vps15* For	5′‐CGATCGATTGAGCACGAG‐3′
*Vps15* Rev	5′‐TGAAGAGCAGGAAGATGTACCA‐3′
*Epg‐3* For	5′‐CGAGTGTCAGAGCCTGGATT‐3′
*Epg‐3* Rev	5′‐CTTTTTGTTGAGGGGCATTG‐3′
*Epg‐5* For	5′‐GCGCCAGGATTAGTAGTCAAG‐3′
*Epg‐5* Rev	5′‐CCAATTGAGGCCAATGAGTT‐3′

Mouse primers for autophagy‐related genes for qPCR experiments (Hsieh et al. 2017).

### Immunohistochemical staining and confocal microscopy

Murine aortas were harvested at sacrifice for histology analysis after undergoing left ventricular puncture and 4% paraformaldehyde (PFA) antegrade perfusion at physiologic pressure. Further fixation was achieved by overnight incubation in 4% PFA at 4°C followed by paraffin embedding and sectioning at 5 μm. After microwave antigen retrieval (HH‐3300, Vector Laboratories Inc., Burlingame, CA), antibodies were bound and detected using VectaStain Elite Kit (Vector Laboratories Inc., Burlingame, CA) and visualized using 3,3′‐Diaminobenzidine (328005000; Acros Organics, Thermo Fisher, NJ, USA). Antibodies for histochemical staining were anti‐Beclin 1 (Abcam, Cambridge, MA, USA), anti‐ATG5 (Abcam), anti‐ATG9 (Abcam), and anti‐LC3 (1:100, 12741S; Cell Signaling Technology, Danvers, MA, USA) (Salmon et al. 2013; Johnston et al. 2014a). Images were acquired using AxioCam Software version 4.6 via 10×, 40×, and 100× objectives and an AxioCam MRc camera (Carl Zeiss Inc., Thornwood, NY). Histology was graded by two blinded reviewers. For elastin grading, 1 corresponded to no degradation, 2 = mild degradation, 3 = moderate degradation, and 4 = severe degradation. For grading, 1 corresponded to no staining, 2 = mild staining, 3 = moderate staining, and 4 = severe staining.

Human and mouse confocal immunoflourescent staining was performed for anti‐Beclin 1 (1:250, ab62557; Abcam) anti‐LC3 (1:100, 12741S; Cell Signaling Technology), smooth muscle actin (SMA) for smooth muscle cells (1:1000, F3777, Sigma Aldrich Corporation, Darmstadt, Germany), DNA was visualized using Hoechst 33342 trihydrochoride trihydrate staining (H3570; Life Technologies, Eugene, OR, USA) (Salmon et al. 2013; Johnston et al. 2014a). Human and mouse tissue were imaged using a Zeiss 710 confocal laser scanning microscope (Zeiss International; Zeiss Germany) at 10× and 63× objectives.

### Mouse aortic smooth muscle cell cultures

Mouse abdominal aortic smooth muscle were isolated and cultured as previously described (Salmon et al. 2012, 2013). Western blot analysis was performed at passage 6 to verify that SM‐actin, SM22, and SM‐MHC were expressed. For siRNA transfections, cells at passages 6–8 were plated in all six wells of a six‐well plate at 1 × 10^5^ cells per well; 24 h later, the cells were transfected with either a single siRNA to mouse Klf4, Klf2, ZFP148, or a non‐targeting control. Cells were allowed to rest for 24 h and then were stimulated by porcine pancreatic elastase (1 unit/mL, E7885; Sigma Aldrich) or IL‐1*β* (10 ng/mL, 401MP; R and D Systems, Minneapolis, MN, USA) and allowed to incubate overnight at 37°C (Salmon et al. 2013). Cells were harvested and RNA was extracted using the TRIzol method and RT‐qPCR was performed as described previously above (Johnston et al. 2013, 2014a,b; Salmon et al. 2013). Efficiency of siRNA transfections has been described previously (Salmon et al. 2013, 2019)

### ChIP analysis

Quantitative chromatin immunoprecipitation assays (ChIP) were performed as described previously (Yoshida et al. 2007, 2008a,b; Salmon et al. 2012, 2013). For qPCR, 2 μL of the 20 μL of extracted DNA was used in 50 cycles of amplification in three steps: 95°C for 15 sec, 55°C for 30 sec, and 68°C for 45 sec. At the end of the amplification cycles, dissociation curves were determined to rule out signal from primer dimers and other nonspecific dsDNA species. Data were normalized to input DNA levels. Experiments were carried out in triplicate and one representative experiment was shown. The size of the qPCR products was confirmed on a 2% agarose gel stained with ethidium bromide. Antibodies include KLF4 (4038; Cell Signaling Technology); KLF2 (ab203591; Abcam), and ZFP148 (ab69933; Abcam) while real‐time PCR primers were designed previously for SMA, SM22*α*, cFOS, and SM‐MHC (Yoshida et al. 2007, 2008b,c). Primers for autophagy genes were described from recent work by Jain and colleagues and are described in Table 4 (Hsieh et al. 2017).

**Table 4:** ChIP primers

BECN1 For	5′‐GCTTCCAATTTGGGTGGATA‐3′
BECN1 Rev	5′‐AATACTGGGCAAGGCATCAT‐3′
LC3B For	5′‐GGGAAGAGCCACAAGATCAG‐3′
LC3B Rev	5′‐TCATTCCCCTTCAGTCCTTG‐3′
ULK1 For	5′‐AACTGTGGGCAGAGCCTAGA‐3′
ULK1 Rev	5′‐GCCATCATGCCTAGTCACCT‐3′
ATG7 For	5′‐GCTCATGACTTCCTGTTGCT‐3′
ATG7 Rev	5′‐CAATGGGCTGTGACTGCAAG‐3′
ATG9 For	5′‐TTTTCCTGGGTGTGTGCTTG‐3′
ATG9 Rev	5′‐ACAAAACACAACATCCCCACT‐3′
CTSD For	5′‐CGTAGAAGCAGCGCATAGTC‐3′
CTSD Rev	5′‐CTCTAGCCCTCTTCTGTGCA‐3′
GABARAPL1 For	5′‐CCTTCTCTGGACGTTTAGCC‐3′
GABARAPL1 Rev	5′‐GATGGACCTCAGGATGTAGGG‐3′
PIK3C3 For	5′‐CTTCTTGCTTCTGTACCCGC‐3′
PIK3C3 Rev	5′‐GGGCGACTCAGTCTATCGG‐3′

Primers for chromatin immunoprecipitation (ChIP) assays for autophagy genes were described previously (Hsieh et al. 2017).

### Statistical methods

Statistical analysis of aortic diameters was performed using GraphPad Prism 7 software (GraphPad Software, La Jolla, CA, USA). Maximal aortic dilation (%) was calculated as [maximal aortic diameter − internal control diameter] ÷ internal control diameter × 100%. The internal control was a small segment of normal proximal abdominal aorta just distal to the renal arteries that was above the proximal extent of the AAA. This section was not perfused with elastase, but it was susceptible to blood pressure changes from volume loss during the harvest as well as expected growth with the animal over time. Values are reported as mean ± standard error of the mean. Aortic dilation between groups was compared using a student's *t*‐test for two sample groups or analysis of variance (ANOVA) for three or more sample groups. Post hoc Tukey correction was applied to determine the significance of individual comparisons with *α *= 0.05 (United States Centers for Disease Control and Prevention, O. o. S. a. P., and the National Center for Injury Prevention and Control, WISQARS 2007). To compare two groups in other samples, a student's *t*‐test was used. Paired data were analyzed by paired student's *t*‐test. Differences between two or more groups versus a control group were analyzed with One‐way ANOVA plus Bonferroni correction for multiple comparisons. Nonparametric data were analyzed by Mann–Whitney *U* test.

## Results

### Beclin and LC3 are up‐regulated in human and murine AAAs

**Figure 1:**
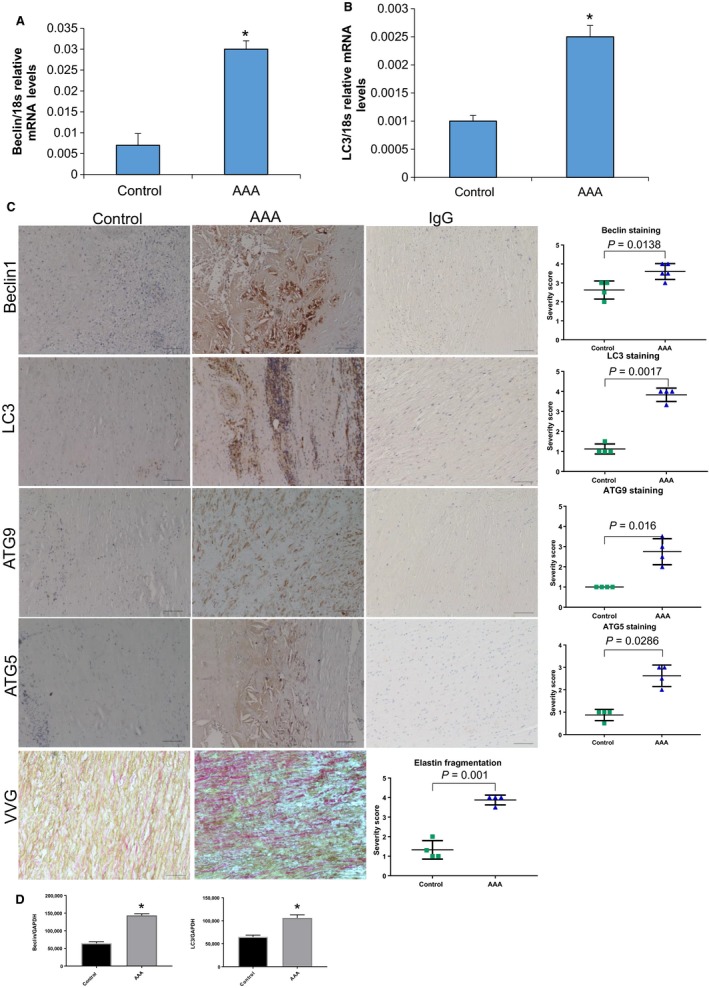
Beclin1 and LC3 are elevated in human abdominal aortic aneurysm (AAA) samples. (A) qPCR analysis of Beclin1 in human control versus AAA tissue (*n* = 10 samples/group). *P = 0.0015 using student's *t*‐test. (B) qPCR analysis of LC3 in human versus AAA tissue (*n* = 10 samples/group). *P = 0.0023 using a student's *t*‐test. (C) Immunohistochemical staining of Beclin1, LC3, ATG5, and ATG9 in human control versus AAA samples (*N* = 6 samples/group). IgG staining as controls for each stain and Verheoff Van Geisen stain to depict aneurysm morphology. Each stain was graded by two individual reviewers and then quantified using a student's *t*‐test. (D and E) Quantification of Western blot of human Beclin and LC3 (*n* = 4/group) using a student's *t*‐test.

Autophagy has recently been demonstrated to be a key factor in the maintenance of endothelial cell wall function (Hsieh et al. 2017). In endothelial cells, autophagy was found to be activated by a conserved *Klf4* mechanism across multiple species and declines with age, the first findings linking autophagy and *Klf4* to age‐related vascular disease (Hsieh et al. 2017). However, an investigation into the role of autophagy activation by *Klf4* in within smooth muscle cells remains to be determined. Secondly, few studies have investigated the role of autophagy in AAA formation; therefore, we sought to establish whether AAA formation also leads to increases in autophagy. Our first goal was to establish whether autophagy is elevated in human AAA. Beclin1 and LC3 levels were measured via qPCR analysis in human AAA versus control healthy donors and were found to increase in human AAA compared to healthy donor controls and compared to AAA samples stained with IgG as a control (Fig. 1A and B; *n* = 10/group). Beclin1 and LC3 staining was also increased in human aneurysm tissue versus controls via immunohistochemistry and was shown to be significantly increased by quantification (Fig. 1C; Beclin *P* = 0.0138 and LC3 *P* = 0.0017; *n* = 10/group). ATG9 and ATG5 were also shown to be significantly elevated by quantification of immunohistochemistry versus control non‐aneurysm tissue (Fig. 1C; ATG9 *P* = 0.016 and ATG5 *P* = 0.286; *n* = 10/group). Elastin fragmentation was measured in the same human samples used for immunohistochemistry to evaluate tissue morphology. Finally, Beclin and LC3 were measured and quantified using Western blot analysis and were found to be elevated versus control human tissue (Fig. 1D; *n* = 4/group). These data suggested that autophagy was elevated in human AAA samples.

**Figure 2:**
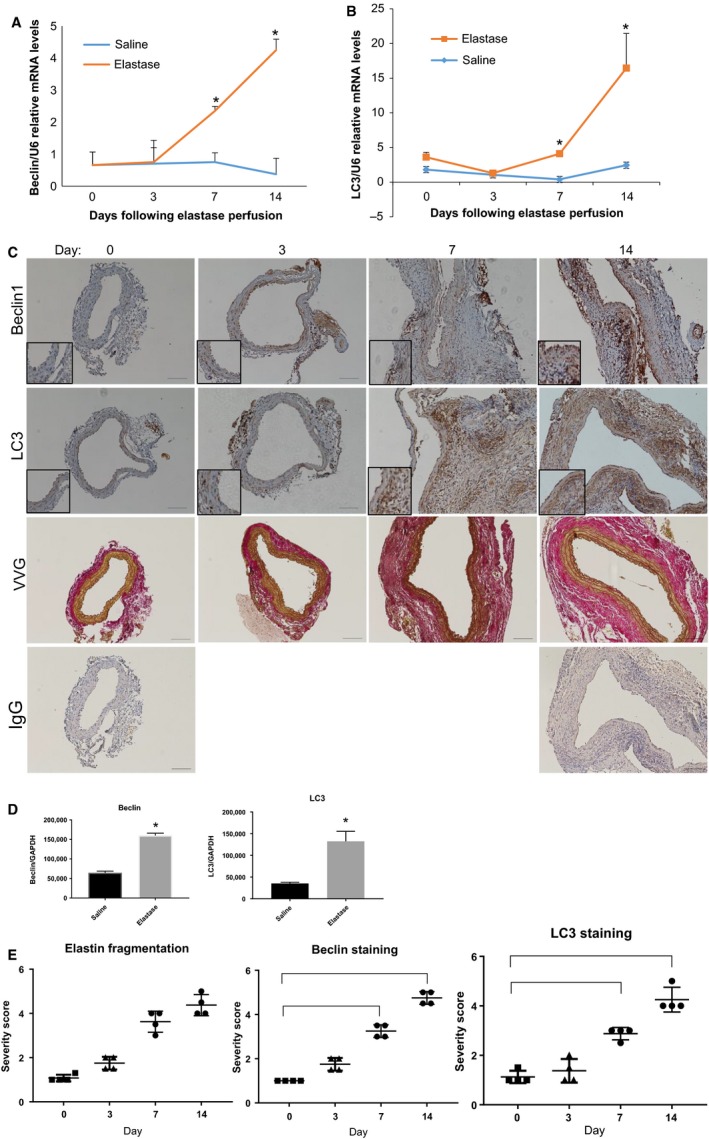
Beclin1 and LC3 are elevated in murine abdominal aortic aneurysm samples. (A) qPCR analysis of Beclin1 in WT elastase versus saline‐perfused aortas (*n* = 6/group) harvested at the indicated time points and RNA was isolated using the TRIzol method. *Indicated *P* < 0.05 over saline controls using student's *t*‐test. (B) qPCR analysis of LC3 in WT elastase versus saline‐perfused aortas as described in part A. (C) Immunohistochemical analysis of Beclin1 and LC3 in WT‐elastase perfused murine aortas harvested at the indicated time points after aneurysm formation. Stains were quantified using two blind reviewers followed by analysis using a student's *t*‐test. Verheoff Van Geisen staining to depict aneurysm morphology and quantified using two blind reviewers followed by a student's *t*‐test. IgG staining as a control at day 0 and 14 in murine WT aortas. (D and E) Quantification of mouse aortas using Western blot analysis followed by a student's *t*‐test (*n* = 4/group).

Following our analysis in human samples, we wanted to determine whether our elastase‐perfusion mouse model would also demonstrate increased levels of autophagy during AAA formation. Therefore, we wanted to determine if we would see increased Beclin1 and LC3 staining in wild‐type murine samples by qPCR, Western blot, and immunohistochemistry (Salmon et al. 2013). Beclin1 and LC3 expressions significantly increased at 7 and 14 days in elastase‐perfused aortas compared to saline‐perfused aortas using qPCR analysis (Johnston et al. 2014a) (Fig. 2A and B; *n* = 6/goup). Following qPCR analysis, we next performed immunohistochemistry and Verheoff Van Geisen stain to assess for aneurysm morphology in our murine model. Staining for Beclin1 and LC3 also increased progressively following elastase perfusion in WT mice as measured by quantification and statistical analysis using a student's *t*‐test (Fig. 2C and E; *n* = 6/goup). Finally, we wanted to determine whether Beclin1 and LC3 were elevated using Western blot analysis. Western blot analysis followed by quantification demonstrated that both Beclin1 and LC3 were elevated in day 14 elastase samples versus control saline‐treated animals at 14 days (Fig. 2D; *n* = 4/group). Our data suggest thus far that autophagy is elevated in human and mouse aortic aneurysm formation.

**Figure 3:**
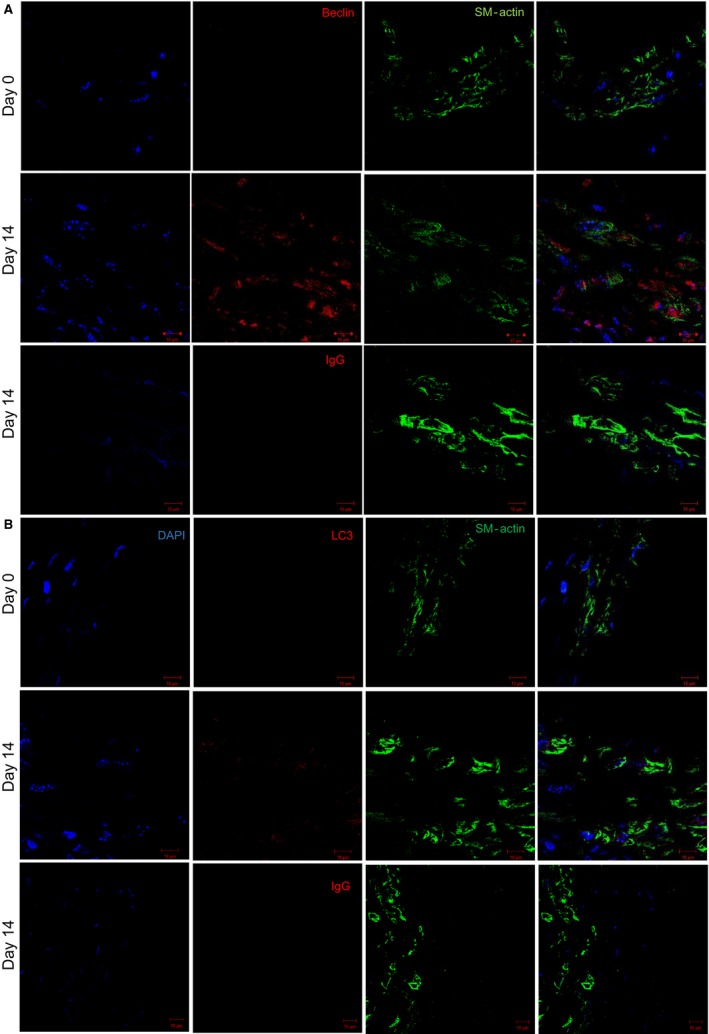
Beclin1 and LC3 localized with SM‐actin+ cells in murine abdominal aortic aneurysm (AAA) samples. (A and B) WT‐elastase perfused murine AAA samples (*n* = 6/group) were stained for either Beclin1 or LC3 (green), SM‐actin (red), and nuclei(blue) followed by imaging using confocal microscopy at day 0 and 14 during murine aneurysm formation. IgG staining as an isotype control for Beclin1 and LC3 in WT aortas at day 14.

Following our determination, that autophagy is elevated in aneurysm formation, we sought to determine what possible cell types autophagy could be activated within. Confocal staining of murine samples at day 0 demonstrated minimal staining for Beclin1 and LC3 (Fig. 3A and B; day 0 staining). Interestingly and consistent with our findings so far, by day 14 staining increased and appeared to be located in cells positive for smooth muscle actin (Fig. 3A and B; day 14 lanes). Furthermore, this staining appeared to be specific for both Beclin1 and LC3 as a IgG control did not exhibit significant staining at day 14 (Fig. 3A and B). Both autophagy factors also co‐localized with CD68+ cells and S100A4+ cells in murine aneurysm tissue; however, we chose not to further pursue these cell types in the in vitro analysis (data not shown). These data suggest that autophagy is elevated during AAA formation and that one cell type autophagy is elevated within is vascular smooth muscle cells.

### Klf4, Klf2, and Zfp148 modulate autophagy‐related genes in phenotypically modulated smooth muscle cells

**Figure 4:**
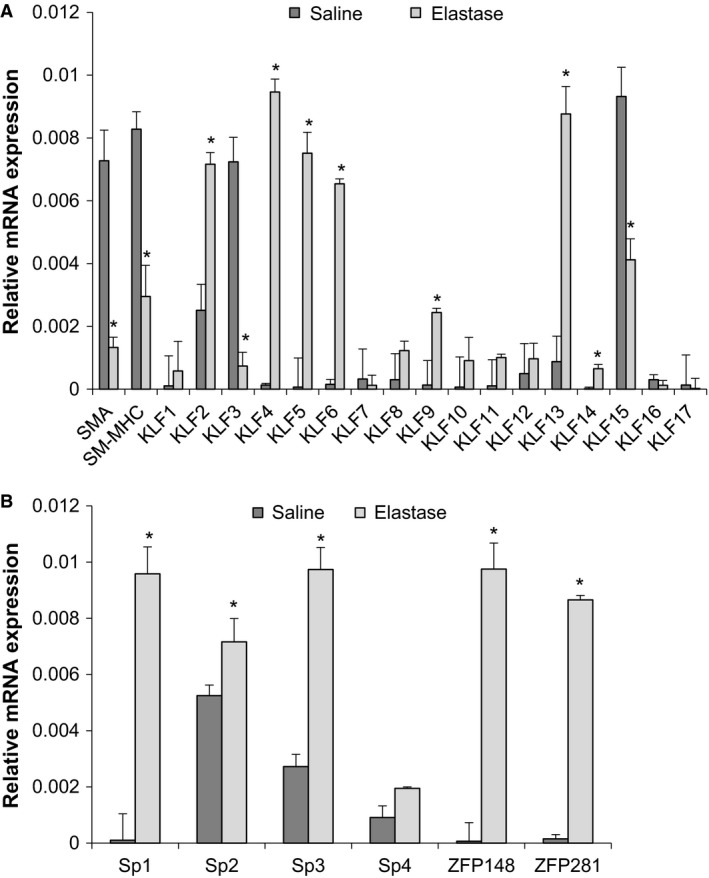
Multiple Kruppel‐like transcription factors are activated in murine abdominal aortic aneurysm (AAA) formation. (A and B) WT elastase versus saline (*n* = 6/group) AAA samples were harvested and RNA was isolated and qPCR was performed for the Kruppel‐like and Sp transcription factor families. Stars indicate significant up‐regulation in elastase AAA samples by student's *t*‐test.

During aneurysm formation, smooth muscle cells undergo a process known as phenotypic modulation. Although this process has been well‐studied, the possible role of autophagy in phenotypically modulated smooth muscle cells during aneurysm formation is not well‐known. Therefore, we hypothesized that another mechanism of phenotypically modulated smooth muscle cells was the activation of autophagy‐related genes during aneurysm formation (Nussenzweig et al. 2015; Ramadan et al. 2017a). We have previously demonstrated that both *Klf4 and Zfp148* are important regulators of smooth muscle cell phenotypic modulation during aneurysm formation; however, we wanted to test to determine whether other Kruppel‐like (Klfs) or zinc‐finger transcription factors could be activated in murine aortic aneurysm formation and could play a role in the activation of autophagy‐related genes in smooth muscle cells. WT elastase or saline‐perfused aortas were harvested at 14 days and subject to qPCR analysis for Klfs and for the Sp/zinc finger transcription factor families. We used SM‐actin and SM‐myosin heavy chain as qPCR controls for phenotypic modulation of smooth muscle cells (Fig. 4A). By qPCR analysis, Klfs 2, 4, 5, 6, 9, 13, and 14 were elevated in WT‐elastase perfused aortas versus saline controls (Fig. 4A). In converse, Klfs 3 and 15 were significantly decreased in elastase‐perfused aortas versus saline controls. Sp family members Sp1, Sp2, Sp3, Zfp148, and Zfp281 were also shown to be elevated in elastase‐perfused WT aortas versus saline controls harvested at day 14 (Fig. 4B). From these studies, we chose to analyze further *Klf4*,* Klf2*, and *Zfp148* since we had previously seen attenuation of AAA formation in murine models using these factors (Salmon et al. 2013, 2019).

**Figure 5:**
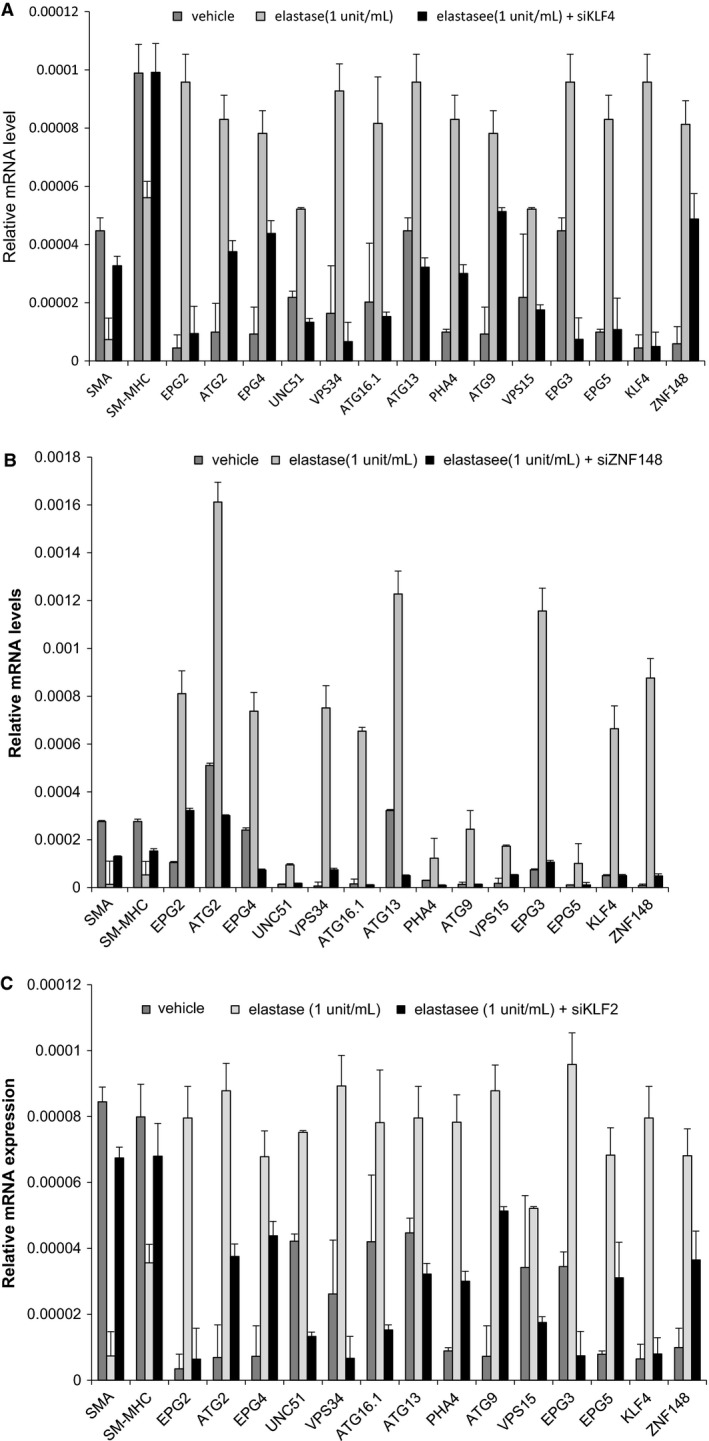
SiKlf4, siKlf2, and siZNF148 inhibit activation of autophagy‐related genes in SM cells following elastase treatment. (A and B) Murine abdominal aortic smooth muscle cells were either treated with: (1) siControl plus vehicle treatment; (2) siControl plus elastase (1 unit/mL); or (3) siKlf4/siZfp148/siKlf2 plus elastase (1 unit/mL) for 24 h and then RNA was isolated and qPCR for the indicated genes was performed. SMA and SM‐MHC exhibited significant down‐regulation in response to elastase treatment by student's *t*‐test. Autophagy genes exhibited significant activation with elastase treatment, which was abrogated with either siKlf4, siKlf2 or siZfp148 by student's *t*‐test.

We hypothesized that *Klf4*,* Klf2*, and *Zfp148* are important for phenotypic modulation and one mechanism of phenotypic modulation during aneurysm formation is by the activation of autophagy. To test this, we extracted RNA and performed qPCR analysis of SMC differentiation marker genes and autophagy genes in murine smooth muscle cells treated with elastase and IL‐1 and either an siControl, siKLF4, siKLF2, or siZFP148. Consistent with our hypothesis, SM‐*α* actin and SM‐MHC both failed to down‐regulate in the smooth muscle cells treated with elastase and siKLF4, siKLF2, or siZFP148 as a control for siRNA effectiveness (Fig. 5A–C). Autophagy genes were also analyzed in vehicle versus elastase versus siKLF4, siKLF2, or siZNF148. Common autophagy genes such as ATG2, ATG16.1, and ATG9 were all activated by treatment with elastase or IL‐1 (Fig. 5A–C and data not shown) (Cao and Klionsky 2007; Yue‐Hong et al. 2012; Hsieh et al. 2017; Vion et al. 2017). Treatment with either siRNA to *Klf4*,* Klf2*, or *Zfp148* and elastase or IL‐1*β* resulted in an abrogation of activated autophagy genes in smooth muscle cells (Fig. 5A–C). These data suggest that *Klf4*,* Klf2*,* and/or Zfp148* might directly regulate the activation of these genes.

**Figure 6:**
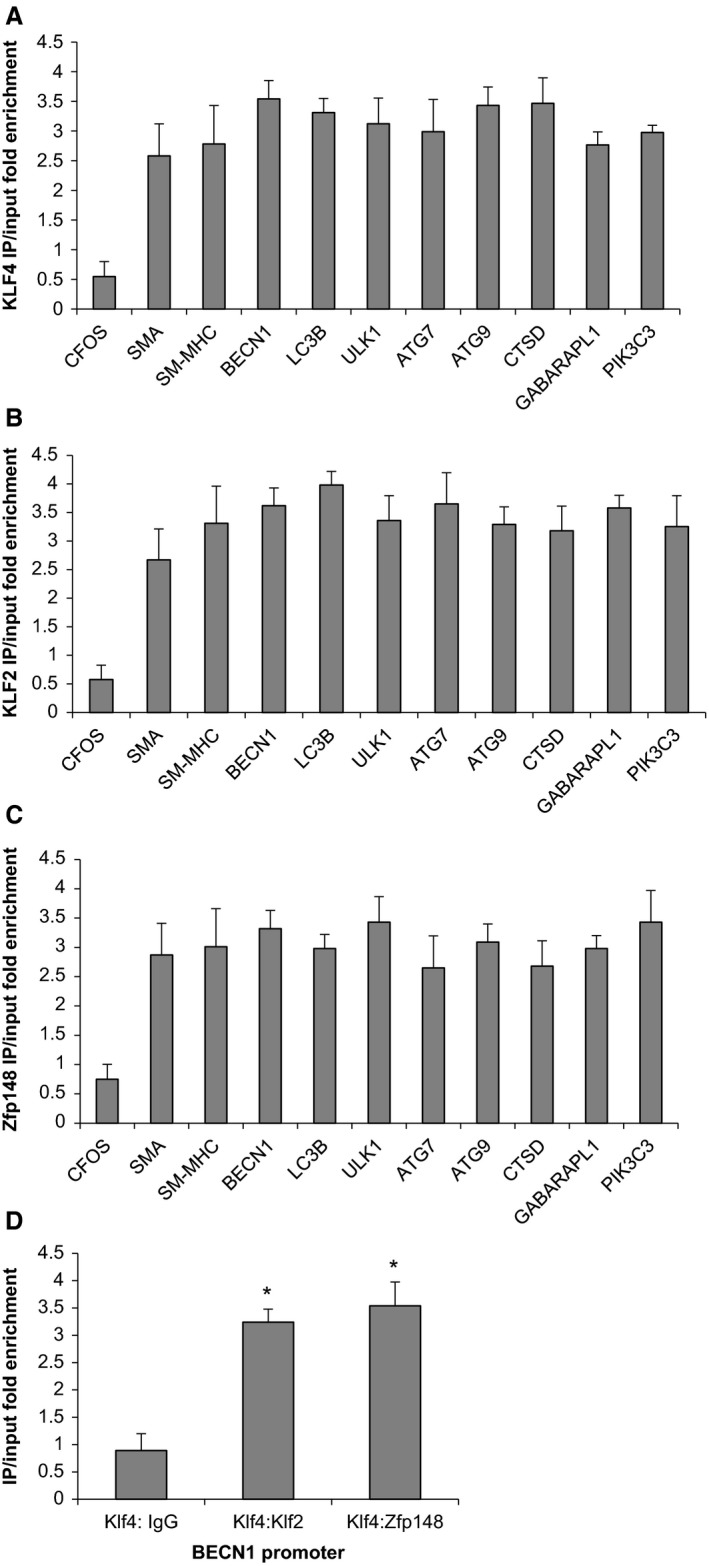
Klf4, Klf2, and Zfp148 bind autophagy genes in smooth muscle cells following treatment with elastase. (A–C) Murine abdominal aortic smooth muscle cells were plated, allowed to grow to confluency then switched to serum‐free media for 3 days. After 3 days, cells were treated with either vehicle or elastase (1 unit/mL) for 24 h followed by harvest of chromatin for chromatin immunoprecipitation (ChIP) analysis. Data are the results of three independent experiments performed in triplicate with one representative experiment depicted. cFOS did not exhibit significant binding following elastase treatment while all other genes analyzed demonstrated significant binding over vehicle control using student's *t*‐test. (D) Murine abdominal aortic smooth muscle cells were cultured as mentioned in part A followed by harvest of chromatin for ChIP analysis. ChIPs were performed first for *Klf4* and then for either IgG, *Klf2* or *Zfp148*. Data are the results of three independent experiments performed in triplicate with one representative experiment depicted. Stars indicate significant binding of sequence ChIP assays by student's *t*‐test.

To determine if *Klf4*,* Klf2*, or *Zfp148* might directly regulate the suppression of SMC marker genes, we performed ChIP assays on chromatin extracted from murine smooth muscle cells 24 h after treatment with elastase (Salmon et al. 2012, 2013). We observed enriched binding of *Klf4*,* Klf2*, or *Zfp148* to the promoters of multiple smooth muscle marker genes, including SM *α*‐actin and SM‐MHC (Fig. 6A–C) (Yoshida et al. 2007, 2008b,c). We performed these assays as controls to ensure that we were seeing binding of these factors to known target genes. There was also enriched binding of several autophagy factors such as Beclin1 and LC3 following treatment with elastase (Fig. 6A–C) (Hsieh et al. 2017). In contrast, there was no enriched *Klf4*,* Klf2*, or *Zfp148* binding to the c‐FOS promoter following elastase perfusion (Fig. 6A–C). To determine if it was possible for these three transcription factors to bind collectively, we performed sequential ChIP analyses. Sequential ChIPs suggested that *Klf4* and *Klf2* could bind to the BECN1 promoter and that *Klf4* and *Zfp148* can also bind to the BECN1 promoter (Fig. 6D). Collectively, these data suggest *Klf4*,* Klf2*, and *Zfp148* play a role in aneurysm formation via direct activation of autophagy genes.

## Discussion

Through these studies, we demonstrated that autophagy is activated during AAA formation and that stimulation with the aneurysm inducing agents, porcine pancreatic elastase and IL‐1*β*, lead to activation of autophagy genes in abdominal aortic smooth muscle cells. Furthermore, we were able to demonstrate that activation of these genes is dependent directly in part upon *Klf4*,* Klf2*, and *Zfp148* binding following treatment with elastase or IL‐1*β* in smooth muscle cells. The present studies are the first to demonstrate that *Zfp148*,* Klf2*, and *Klf4* contribute to the activation of autophagy in smooth muscle cells as part of AAA formation. Previous studies have demonstrated that KLFs are essential to the activation of autophagy during nematode lifespan and suggest that *Klf4* is essential for augmenting autophagy and improving vessel function in the endothelium (Hsieh et al. 2017). Jain et al., further suggest that *Klf4* levels naturally decline in the endothelium with age and that this decline is linked with the declining function of endothelial cells (Hsieh et al. 2017). These studies point to the activation of autophagy in endothelial cells being a positive cellular response that is activated transcriptionally by *Klf4* (Liu et al. 2015). In contrast, our studies suggest that augmentation of autophagy in smooth muscle cells by *Klf4* is linked with the transition of smooth muscle cells to a more proliferative, migratory, and synthetic state, a maladaptation common in vascular diseases such as AAA or atherosclerosis (Ramadan et al. 2017a,b). Autophagy has been linked to having either positive or negative effects in smooth muscle cells depending on the treatment therapy and we are reporting an incidence where activation of autophagy appears to have negative effects for smooth muscle cells during aneurysm formation (Ramadan et al. 2015, 2017a,b).

Interestingly, these paradoxes of differential activity in the same KLF family member appear to be common in vascular disease pathology. *Klf4* has been shown by our laboratory to be crucial to phenotypic modulation of smooth muscle cells in both aneurysm and by other laboratories in atherosclerotic lesion formation (Feinberg et al. 2005; Hamik et al. 2007; Salmon et al. 2013; Shankman et al. 2015), which would suggest that *Klf4* is an activator of vascular disease formation. However, in endothelial cells through the present investigation of endothelial function and in macrophages where macrophage‐specific *Klf4* deletion was found to augment atherosclerotic lesion formation, *Klf4* would be considered protective against vascular disease formation (Feinberg et al. 2005; Hamik et al. 2007). Recent unpublished studies by our laboratory also suggest that Klf2 functions in smooth muscle cells to induce phenotypic modulation of smooth muscle cells while published studies suggest that *Klf2* functions to protect endothelial barrier function. Therefore, these multiple paradoxical roles for KLF transcription factors appears to be common since both *Klf2* and *Zfp148* also appears to have multiple different functions dependent upon cell type in vascular disease formation (Sen‐Banerjee et al. 2005; Das et al. 2006; Libby et al. 2006; Atkins et al. 2008; Sayin et al. 2014). Therefore, more detailed studies into the role of the KLF family during AAA formation is warranted since currently investigations are limited as to the multiple complex roles of KLFs during aneurysm disease.

The paradoxical effects of these transcription factors in multiple different cell types also make the KLF family a difficult pharmacologic target. Aneurysm disease remains one of several vascular diseases that currently has no medical treatment therapy; therefore, much of the current work in the field is focused on finding a viable medical treatment option beyond surgical intervention. Since KLFs have multiple functions in various cell types and would be a difficult viable medical target therapy for AAA disease, perhaps targeting autophagy directly would be beneficial to inhibiting AAA formation and/or progression? Recent studies would suggest that autophagy inhibition does not affect aneurysm formation as inhibition of autophagy using chloroquine, a common autophagy inhibitor, did not affect aneurysm incidence or aortic diameter using the ApoE^−/−^ Angiotensin II mouse model of AAA (Ramadan et al. 2015, 2017b). However, in converse, separate studies looking at the role of rapamycin, everolimus, and resveratrol, all considered other possible autophagy inhibitors, were all seen to inhibit aneurysm formation (Ramadan et al. 2017a). Therefore, there is still considerable debate concerning the merits of targeting autophagy specifically clinically in human AAA disease.

In considering the possible limitations of this study, we are unable to ascertain the direct effect of autophagy on AAA formation without the creation of smooth muscle, endothelial, macrophage, neutrophil, and T‐cell specific conditional knock‐outs. These studies would delineate specifically the merits of specifically targeting autophagy during AAA formation. Furthermore, Shankman et al. (2015) have recently developed smooth muscle specific lineage tracing mice to determine the effects of cell‐specific eliminate of factors on smooth muscle phenotypic fate. Combining cell lineage studies with cell‐specific autophagy deletions would help to directly determine the causal roles of autophagy during AAA formation. However, many of the inflammatory cell conditional knock‐outs are cross reactive for several different cell types and would confound the results of cell‐specific deletion during aneurysm formation in mouse models. It would be interesting to postulate whether cell‐specific removal of key autophagy factors, such as ATG5, ATG6 or ATG12, would result in a phenotype unique to aneurysm disease. In summary, these studies represent only one possible mechanism for *Klf4*,* Klf2*, and *Zfp148* as activators of autophagy during AAA formation and lead further credence to the importance of autophagy, *Klf4*,* Klf2*, and *Zfp148* in broad significance for the overall aneurysm phenotype. Future studies by our laboratory and others are needed to directly test the roles cell‐specific elimination of autophagy plays in the determination of aneurysm formation and rupture.

## Conflict of Interest

None declared.
